# Agonistic Behavior and Energy Metabolic Responses of *Eriocheir sinensis* Under Thermal Stress with Insights from Untargeted Metabolomics

**DOI:** 10.3390/ani16213440

**Published:** 2026-10-31

**Authors:** Yanan Liu, Lili Xu, Qun Liu, Sudong Xia, Xuying Jia, Yan Sun, Wenli Zhou, Dan Zhang

**Affiliations:** 1 Tianjin Key Laboratory of Aqua-Ecology and Aquaculture, Fisheries College, Tianjin Agricultural University, Tianjin 300384, China; liuyanan020802@163.com (Y.L.); panbakong358@163.com (L.X.); tjcadcliuq@163.com (Q.L.); xsd20022003@126.com (S.X.); jiaxuying@tjau.edu.cn (X.J.); sunyan19850409@aliyun.com (Y.S.); wenlizhou@tjau.edu.cn (W.Z.); 2 Key Laboratory of Smart Breeding (Co-Construction by Ministry and Province), Ministry of Agriculture and Rural Affairs, Tianjin Agricultural University, Tianjin 300384, China; 3 Animal Disease Prevention and Control Center of Tianjin, Tianjin 300402, China

**Keywords:** *Eriocheir sinensis*, thermal stress, swimming behavior, agonistic behavior, energy metabolism, untargeted metabolomics

## Abstract

The Chinese mitten crab (*Eriocheir sinensis*) is an important freshwater aquaculture species in China, but high summer temperatures may disrupt its behavioral patterns and energy metabolism. In this study, male *E. sinensis* were maintained at 25 °C or 35 °C, and behavioral tracking, histological observation, biochemical assays, RT-qPCR, and untargeted metabolomics were combined to investigate the effects of thermal stress on swimming and aggressive behaviors and their associated metabolic responses. For swimming behavior, compared with 25 °C, crabs at 35 °C showed decreases in total moving distance, average speed, and the proportions of slow, medium, and fast-speed movement, together with a significant increase in inactive time (*p* < 0.05). For agonistic behavior, compared with 25 °C, both the overall proportion of agonistic intensity and the proportion of strong fighting decreased significantly at 35 °C (*p* < 0.05). According to agonistic behavior data, the crabs were classified as winners or losers, generating four groups: T25W, T25L, T35W, and T35L. Non-contact behaviors decreased significantly in both winners and losers, whereas contact behaviors also decreased significantly in winners (*p* < 0.05). Histopathological analysis revealed that, at 35 °C, *E. sinensis* exhibited disorganized muscle fibers and structural damage to the gills, including swelling of the distal lamellar tips and morphological abnormalities in epithelial and pillar cells. With respect to energy metabolism, muscle glycogen in winners was significantly higher than that in losers at both temperatures, whereas haemolymph lactate concentrations were significantly higher in winners only at 25 °C. Haemolymph glucose concentrations were significantly higher in winners at 25 °C but in losers at 35 °C (*p* < 0.05). Compared with 25 °C, exposure to 35 °C significantly decreased muscle succinate dehydrogenase (SDH) activity in both winners and losers and increased gill citrate synthase (CS) activity in winners. In losers, muscle pyruvate kinase (PK) activity decreased nonsignificantly; lactate dehydrogenase (LDH) activity increased (*p* < 0.05). Under 35 °C conditions, significant upregulation of *AMPK*, *Sestrin*, and *SIRT1* expression and significant downregulation of *mTOR* expression (except in losers’ muscle) were exhibited in both muscle and gill tissues (*p* < 0.05). Untargeted metabolomics identified 297, 301, 484, and 393 differential metabolites in comparisons in T25W vs. T25L, T35W vs. T35L, T35W vs. T25W, and T35L vs. T25L, respectively. These differential metabolites were mainly associated with amino acid, nucleotide, and lipid metabolism, aminoacyl-tRNA biosynthesis, ABC transporters, and neuroactive ligand–receptor interaction. Collectively, acute exposure to 35 °C suppressed swimming and agonistic behavior, with this behavioral inhibition being associated with tissue damage and regulation of energy metabolism. This study provides candidate indicators and a scientific basis for the early assessment and monitoring of thermal stress in *E. sinensis* aquaculture.

## Simple Summary

Extremely high temperatures during summer severely constrain the sustainable development of the *Eriocheir sinensis* farming industry, while behavior represents one of the most direct indicators of the responsiveness to environmental change. We compared male crabs at 25 °C and 35 °C to examine changes in movement, fighting, tissue condition, and energy use. At 35 °C, crabs moved less, remained inactive longer, and engaged in fewer and less intense fights. Thermal stress also damaged muscle and gill tissues and altered how energy was stored and used. These findings may support early recognition and improved management of thermal stress in *E. sinensis* farming.

## 1. Introduction

The Chinese mitten crab (*Eriocheir sinensis*) is one of the most widely cultured crab species in China and is of considerable economic importance to freshwater aquaculture [1]. Water temperature is an important ecological factor affecting the growth and metabolism of *E. sinensis* [2,3,4,5]. Against the backdrop of climate warming and increasingly frequent extreme temperature events, persistently high summer temperatures in major aquaculture regions expose cultured *E. sinensis* to thermal stress, potentially resulting in substantial production losses [6]. As an ectotherm, *E. sinensis* is highly dependent on ambient temperature for the regulation of its metabolic processes and energy allocation. Under thermal stress, *E. sinensis* may experience an imbalance between energy demand and supply, resulting in an energy deficit that may threaten its survival, compromise tissue integrity, and disrupt energetic and physiological homeostasis [5,7]. Similar to other crustaceans, *E. sinensis* is highly susceptible to stress and mortality under high-temperature conditions [8]. Thermal stress damaged the intestinal mucosa of *Pandalus latirostris* and resulted in 100% mortality after 120 h, suggesting that thermal stress-induced damage may progress from sublethal injury to death [9]. Kong et al. showed that acute thermal stress disrupted physiological homeostasis and progressively impaired metabolic function in *Sinonovacula constricta* [10]. Taken together, these findings indicate that thermal stress adversely affects the survival, growth, and metabolism of crustaceans, thereby constraining the development of crustacean aquaculture and diminishing its economic benefits.

Behavioral responses are among the most direct indicators of how aquatic animals perceive and respond to environmental changes. In crustaceans, swimming behavior reflects locomotor capacity and responsiveness to environmental stimuli [11,12]. As a characteristic and ecologically important behavior of crustaceans, agonistic behavior enables individuals to effectively acquire or retain food, shelter, space, and other resources. In *E. sinensis* aquaculture, video-based monitoring of agonistic behavior could help identify changes in competitive activity during hot periods and support feeding management and temperature control [13]. In aquaculture, assessments of environmental stress have traditionally relied primarily on physiological and biochemical indicators, whereas behavioral endpoints have received relatively limited attention [14]. Previous research showed that elevated temperature altered the intensity, duration, and frequency of agonistic behavior in *Portunus trituberculatus* and changed the energy demands associated with fighting [13]. Similarly, some behavioral disturbances remained detectable during post-stress recovery in the blue mussel *Mytilus edulis* [15]. Therefore, characterizing the behavioral responses of *E. sinensis* to thermal stress may provide direct indicators for assessing its functional condition under high-temperature conditions.

Untargeted metabolomics provides a powerful approach for comprehensively characterizing the metabolic responses of *E. sinensis* to thermal stress [16]. Previous studies have applied untargeted metabolomics to characterize the metabolic responses of *E. sinensis* to air-exposure stress and *Litopenaeus vannamei* to high-temperature stress [17,18]. Although previous studies have characterized the metabolomic responses of crustaceans to environmental stress [13,19], the metabolomic changes associated with behavioral responses in *E. sinensis* under thermal stress remain poorly understood. Integrating behavioral observations with untargeted metabolomics may therefore help identify metabolic changes associated with these behavioral responses.

Global climate change exposes *E. sinensis* to high water temperatures during summer, potentially altering its behaviors and associated energy metabolism. To investigate these effects, we quantified swimming and agonistic behavioral outcomes in male crabs exposed to 25 °C and 35 °C and systematically examined muscle and gill histology, energy substrates, metabolic enzyme activities, energy-sensing gene expression, and metabolomic analysis of muscle. These findings deepen our understanding of the metabolic mechanisms underlying behavioral impairment under thermal stress and provide a behavioral basis for managing summer thermal stress in *E. sinensis* aquaculture.

## 2. Materials and Methods

### 2.1. Experimental Animals and Thermal Stress Treatment

A total of 150 male *E. sinensis* of similar body size, with a mean body weight of 47.13 ± 1.36 g, were collected from Qilihai Wetland, Tianjin, China, on 20 July 2025. The crabs were transported to the laboratory and acclimated for 7 days at 25 °C in water with a salinity of 3‰ under a 12 h light:12 h dark photoperiod. During acclimation, all crabs were fed the same commercial pelleted diet once daily at 17:00 at 5% of their body weight. Crabs were fasted for 24 h before behavioral testing and were not fed during the experiment. Only healthy, intermolt crabs with intact appendages and normal feeding behavior were selected. Each crab was marked on the carapace with white acrylic paint for individual identification. Crabs were randomly assigned to a control group maintained at 25 °C and a thermal-stress group exposed to 35 °C. Preliminary trials showed no significant differences in the assessed responses between 25 and 30 °C, whereas mortality occurred at 40 °C. Therefore, 35 °C was selected as the thermal-stress temperature, with additional support from previous studies in *E. sinensis* [20,21]. For thermal stress, the water temperature was increased from 25 °C at 2 °C/h until it reached 35 °C. After reaching 35 °C, the temperature was maintained for 10 min before behavioral recording began. Crabs were fasted for 24 h before the experiment and were not fed during the experiment. Animal experiments were approved by the Experimental Animal Ethics Committee under approval no. 2025LLSC64.

### 2.2. Behavioral Recording and Analysis

#### 2.2.1. Swimming Behavior Recording and Analysis

Swimming behavior was recorded in a transparent observation tank (30 cm × 25 cm × 15 cm). The video-capture system consisted of an industrial infrared camera (HIKVISION, DS-2CD864, Hikvision Digital Technology, Hangzhou, China; infrared wavelength, 850 nm), a monitor (PHILLIP, 233i, MMD Electronics Technology, Shanghai, China), and heating rods. The camera was fixed 0.4 m above the experimental tank, and the water depth was maintained at 10 cm throughout the experiment. Sixteen crabs were assigned to each temperature group (32 crabs in total) and tested individually, with swimming behavior recorded continuously for 40 min following a 10-min stabilization period at the target temperature. Recordings were analyzed using the Tracking Master (V4.4.2,Vanbi Intelligent Technology, Shanghai, China) behavioral tracking system, with the entire tank defined as the observation arena. Total distance moved, mean swimming speed, instantaneous speed, and the time spent in slow, medium-speed, fast, and inactive states were calculated to characterize swimming behavior.

#### 2.2.2. Agonistic Behavior Recording and Quantification

A total of 80 crabs were used for agonistic interactions, with 40 crabs at each temperature arranged into 20 pairs in the observation tank described above. The two opponents were placed on opposite sides of a separator board and allowed to settle in isolation for 10 min. Commercial feed pellets were ground, and 0.5 g of the resulting powder was mixed with 5 mL of water to prepare a feed suspension, corresponding to a feed-to-water preparation ratio of 1 g:10 mL. For each trial, a 5 mL aliquot of the suspension was slowly introduced into the center of the arena as the separator board was raised. The same preparation ratio and application volume were used at both temperatures to provide a standardized food-associated stimulus. The ensuing interactions were recorded for 45 min and saved to the host computer for subsequent analysis. To promote consistent and objective scoring, all recordings were assessed by a single observer blinded to the temperature treatments, using the same predefined behavioral criteria. Scoring consistency was not separately quantified. Each contest consisted of discrete agonistic behaviors defined in Table 1. These behaviors were divided into non-contact behaviors (move to, move away, and cheliped display) and contact behaviors (strike, grasp, push, and climb on) Behavioral frequency was defined as the number of times a behavioral element was performed during a contest. Fight intensity was classified as very weak, weak, moderate, or strong according to the interaction and physical-contact criteria listed in Table 2. The proportion assigned to each intensity level was calculated relative to the full observation period; periods without agonistic interaction were not assigned to any intensity level. The winner was defined as the crab that elicited continuous retreat by its opponent or successfully climbed onto the opponent; the other crab was classified as the loser. According to the contest outcome, crabs were assigned to four groups: winners at 25 °C (T25W), losers at 25 °C (T25L), winners at 35 °C (T35W), and losers at 35 °C (T35L). Behaviors performed by winners and losers were quantified separately.

### 2.3. Tissue Collection and Sample Preparation

Immediately after the agonistic trials, crabs were removed with minimal disturbance and placed in cold water to induce general anesthesia. Muscle, gill, and hemolymph samples were then collected on ice. Muscle and gill tissues were used for histology, enzyme activity assays, and RT-qPCR; muscle was also used for glycogen determination and untargeted metabolomics, and hemolymph for glucose and lactate assays. For energy-substrate measurements, enzyme activity assays, and RT-qPCR, eight crabs per group were sampled, with corresponding samples pooled in pairs to form four biological samples (*n* = 4). Each winner pool contained samples from two winners, while its matched loser pool contained samples from their corresponding opponents in the same two contests. Thus, four matched pairs of pooled biological samples were obtained within each temperature treatment. For untargeted metabolomics, muscle samples from ten crabs per group were pooled in pairs to form five biological samples (*n* = 5). Tissue samples for these analyses were flash-frozen in liquid nitrogen and stored at −80 °C. Hemolymph was collected from the arthrodial membrane at the base of the last pereiopod, centrifuged at 6000 rpm for 10 min at 4 °C, and the supernatant was stored at −80 °C.

### 2.4. Histological Analysis

For histological analysis, separate muscle and gill specimens were fixed in Davidson’s AFA solution for 24 h, with the fixative volume exceeding ten times the tissue volume. Before section preparation, samples were transferred to 70% ethanol for 36 h and stored at 4 °C. Paraffin embedding and section preparation were performed by Nanjing Zhongding Biotechnology Co., Ltd. (Nanjing, China). Tissue sections were cut at 5–6 μm, stained with hematoxylin and eosin (HE), and mounted with neutral balsam. Sections were examined and photographed under a 10× objective using a Leica biological microscope (Leica Microsystems GmbH, Wetzlar, Germany) and NIS-Elements F 4.0 software (Nikon Corporation, Tokyo, Japan). Changes in gill and muscle structure were compared between temperature groups. Histological assessment was qualitative and provided complementary morphological observations for the behavioral and energy-metabolic analyses. Representative micrographs were selected for presentation.

### 2.5. Determination of Energy Substrates and Enzyme Activities

Muscle glycogen was measured by the anthrone–sulfuric acid colorimetric method using kit A043-1-1, hemolymph glucose by the glucose oxidase–peroxidase method using kit F006-1-1, and hemolymph lactate by the NBT colorimetric method using kit A019-2-1 (Nanjing Jiancheng Bioengineering Institute, Nanjing, China). The final optical densities were measured at 620, 505, and 530 nm for glycogen, glucose, and lactate, respectively, using an automatic microplate reader (Synergy2, BioTek, Winooski, VT, USA). Enzyme activities were measured colorimetrically using kits from the same manufacturer: citrate synthase (CS, A108-1-1), succinate dehydrogenase (SDH, A022-1-1), malate dehydrogenase (MDH, A021-2-1), Na^+^/K^+^-ATPase (A016-1-1), hexokinase (HK, A077-3-1), pyruvate kinase (PK, A076-1-1), phosphofructokinase (PFK, A129-1-1), and lactate dehydrogenase (LDH, A020-2-2). Samples were processed according to the corresponding kit instructions. Each pooled biological sample was assayed in three technical replicates. Glycogen content was expressed as mg/mg protein, hemolymph glucose as μmol/mg protein, and hemolymph lactate as mmol/mg protein. Enzyme activities in muscle and gill tissues were expressed as U/mg protein.

### 2.6. Gene Expression Analysis by RT-qPCR

Total RNA was extracted from approximately 50 mg of gill and muscle tissue powder using TRIzol reagent (Aidlab, Beijing, China). The concentration and purity of RNA were measured with a NanoDrop 2000 spectrophotometer (Thermo Fisher Scientific, Wilmington, DE, USA). RNA integrity was checked by 1.2% agarose gel electrophoresis. Then, 1 μg of total RNA was reverse-transcribed into first-strand cDNA using the PrimeScript™ RT Reagent Kit (Takara Bio Inc., Kusatsu, Japan) according to the manufacturer’s instructions. The qPCR was performed using an Applied Biosystems 7500 Real-Time PCR System (Thermo Fisher Scientific, Waltham, MA, USA) with 2× SYBR Green qPCR Mix (Aidlab Biotechnologies Co., Ltd., Beijing, China). Each reaction was carried out in a final volume of 20 μL. The reaction mixture contained 10 μL of SYBR Green mix, 0.8 μL of each primer at 10 μM, 2 μL of 5-fold diluted cDNA, and 6.4 μL of nuclease-free water. The qPCR cycling conditions included an initial denaturation at 95 °C for 30 s, followed by 40 cycles of 95 °C for 5 s and 60 °C for 34 s. Each biological sample was analyzed in three technical replicates. The target genes included *AMPK*, *Sestrin*, *SIRT1*, and *mTOR*. *β-actin* was used as the internal reference gene. Relative mRNA expression levels were calculated using the 2^−ΔΔCt^ method. The qPCR primer sequences are listed in Table 3.

### 2.7. Untargeted Metabolomics Analysis

Approximately 50 mg of each sample was mixed with 400 μL of pre-cooled methanol–water (4:1, *v*/*v*) containing L-2-chlorophenylalanine as an internal standard, homogenized under low-temperature conditions, and sonicated in an ice–water bath. After protein precipitation at −20 °C for 30 min, the extracts were centrifuged at 13,000× *g* for 15 min at 4 °C, and the supernatants were collected. Equal aliquots of the sample extracts were pooled to prepare quality-control (QC) samples. Samples were analyzed in randomized order, with QC samples included at the beginning, middle, and end of the analytical sequence. Metabolites were separated using a Thermo Vanquish Flex UPLC (Thermo Fisher Scientific, Germering, Germany) system equipped with an ACQUITY UPLC HSS T3 column (100 mm × 2.1 mm, 1.8 μm; Waters). Mobile phase A was 5 mmol/L ammonium acetate plus 5 mmol/L acetic acid in water, and mobile phase B was acetonitrile. The gradient was 0–0.8 min, 2% B; 0.8–2.8 min, 2–70% B; 2.8–5.0 min, 70–90% B; 5.0–5.5 min, 90–100% B; 5.5–7.5 min, 100% B; 7.5–7.6 min, 100–2% B; and 7.6–10.0 min, 2% B. The flow rate was 0.35 mL/min, the injection volume was 4 μL, and the column temperature was 40 °C. Metabolites were detected using an Orbitrap Exploris 120 high-resolution mass spectrometer (Thermo Fisher Scientific) in positive and negative electrospray ionization modes. The source temperature was 350 °C; spray voltage was +3.8 kV in positive mode and −3.4 kV in negative mode; sweep, auxiliary, and sheath gas flows were 1, 15, and 50 Arb, respectively. Data were acquired using full-scan and data-dependent acquisition (DDA) modes over 70–1050 Da. Full-scan resolution was 60,000, and the four most intense ions with signal intensity above 5000 were selected for MS/MS at a resolution of 15,000; dynamic exclusion was 4 s. Raw LC-MS files were converted to mzXML format and processed using XCMS [22], CAMERA [23], and metaX [24] in R (v4.0). Data processing included peak detection, peak grouping, retention-time correction, isotope annotation, and adduct annotation. Features with more than 50% missing values in QC samples or more than 80% missing values in experimental samples were excluded. Data were normalized using probabilistic quotient normalization, and missing values were imputed using the k-nearest neighbors method. Metabolites with a QC coefficient of variation below 30% were retained. Metabolites were annotated using MetDNA3, with a mass tolerance below 10 ppm, an MS/MS spectral matching score > 0.8, and a retention-time tolerance < 25 s. Isotopic patterns and an in-house MS/MS spectral library were used to further assess the annotations. PCA was performed using metaX, and PLS-DA was performed using ropls to assess sample clustering and group separation. VIP scores were obtained from the PLS-DA model. Model performance was assessed using sevenfold cross-validation and 200 permutation tests, with R^2^ and Q^2^ used to describe model fit and predictive performance, respectively [25]. Pairwise differences were evaluated using *t*-tests on log2-transformed feature abundances. Differential metabolites were screened using three simultaneous criteria: a PLS-DA VIP score ≥ 1, a fold change ≥ 1.2 or ≤1/1.2, and an unadjusted *p* < 0.05. FDR correction was not applied, and the selected metabolites were considered exploratory candidates. Hierarchical-clustering heat maps were generated using pheatmap. KEGG pathway enrichment was evaluated using hypergeometric tests, and pathways with an unadjusted *p* < 0.05 were considered enriched.

### 2.8. Statistical Analysis

All data are presented as mean ± standard deviation (SD). Statistical analyses were performed using SPSS 26.0, and figures were generated using GraphPad Prism 11. Swimming variables, fighting-intensity proportions, and agonistic-behavior frequencies were analyzed using generalized linear models in SPSS 26.0 with the program’s default settings. Temperature was included as the fixed factor for swimming variables and fighting intensity. Temperature, fighting outcome, and their interaction were included as fixed effects for agonistic-behavior frequencies. The confidence level was set at 95%. Supplementary analyses of non-contact and contact behavioral frequencies used negative-binomial generalized linear mixed-effects models with temperature, fighting outcome, and their interaction as fixed effects and contest identity as a random intercept to account for pairing (Table S1). Energy-substrate levels and muscle enzyme activities and gene-expression levels were analyzed by two-way ANOVA, with temperature (25 °C and 35 °C) and fighting outcome (winner and loser) as fixed factors and their interaction included in each model. These ANOVA models assumed normally distributed residuals and corresponded to linear models with an identity link. When a significant interaction was detected, simple-effect comparisons were performed using Tukey’s multiple-comparison test. Gill enzyme activities and gene-expression levels were analyzed by two-way ANOVA followed by Tukey’s multiple-range test. Normality and homogeneity of variance were evaluated using the Shapiro–Wilk and Levene tests, respectively. Differences were considered significant at *p* < 0.05.

## 3. Results

### 3.1. Swimming Behavior of E. sinensis Under Thermal Stress

**Figure 1:**
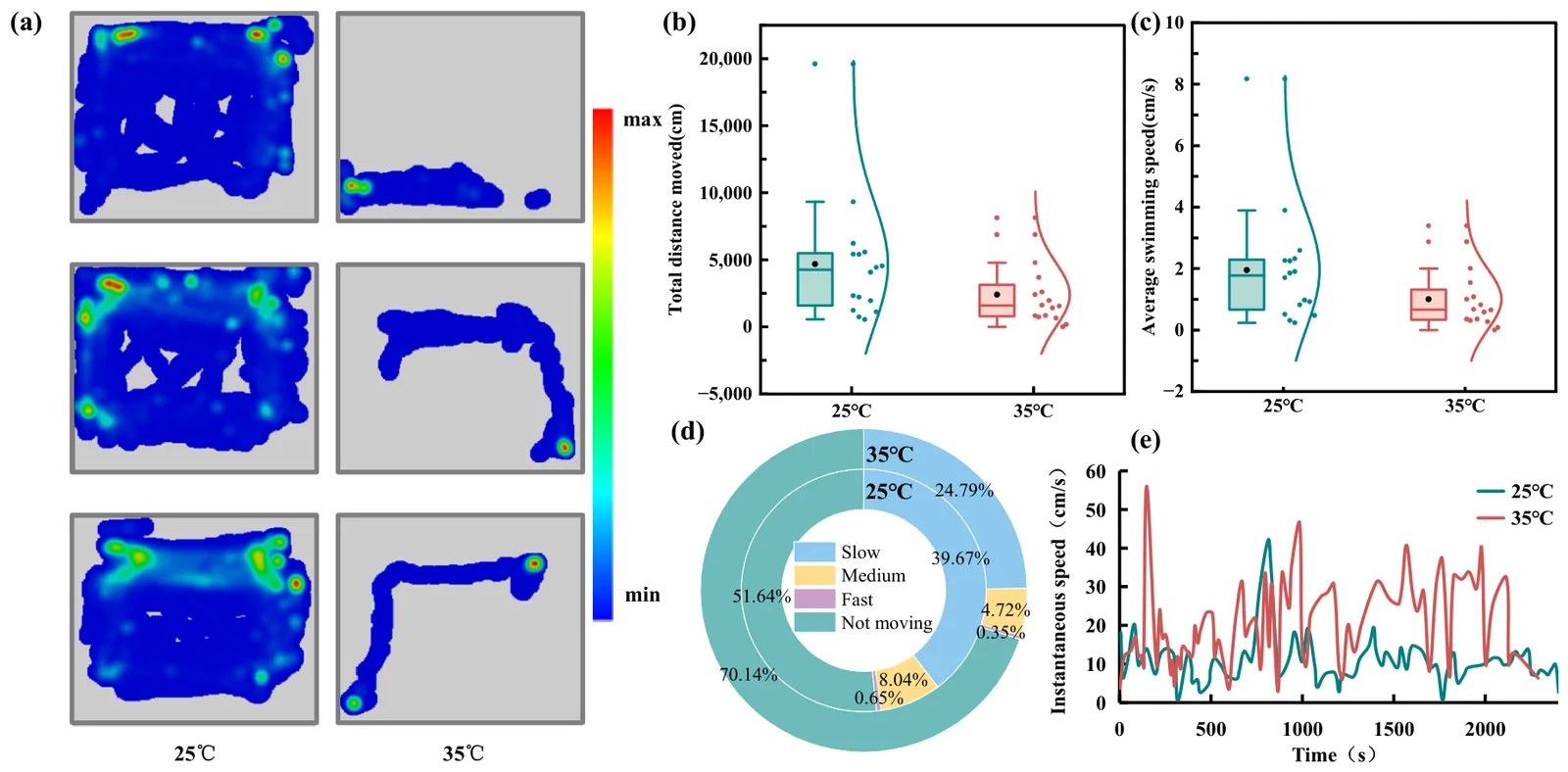
Swimming behavior of *E. sinensis* under different temperature conditions. (**a**) Heat maps showing movement trajectories (the color scale indicates the relative spatial concentration of tracked positions, with blue representing low values and red representing high values); (**b**) total distance moved; (**c**) mean swimming speed; (**d**) time proportions of slow, medium-speed, fast, and inactive states; (**e**) instantaneous-speed profiles. In (**b**,**c**), boxes represent the interquartile range (25th–75th percentiles), horizontal lines within the boxes indicate medians, and black diamonds indicate means. Whiskers extend to the most extreme observations within 1.5 times the interquartile range from the box edges. Each dot represents one individual, and the adjacent curves show the estimated probability density distributions. Data are presented as mean ± SD (*n* = 16). Differences between the two temperature groups were analyzed using generalized linear models.

The heat maps showed that crabs at 25 °C moved throughout most of the observation arena and formed several high-activity regions. At 35 °C, movement tracks were shorter and concentrated within a smaller area, with fewer high-activity regions. Total distance moved decreased from 4673.14 cm at 25 °C to 2393.99 cm at 35 °C, a reduction of approximately 48.77% (Figure 1b). Mean swimming speed decreased from 1.95 cm/s to 1.00 cm/s, corresponding to a reduction of approximately 48.72% (Figure 1c). At 25 °C, crabs spent 39.67% of the observation period moving slowly, 8.04% moving at medium speed, 0.65% moving rapidly, and 51.64% in the inactive state (Figure 1d). At 35 °C, the corresponding proportions were 24.79%, 4.72%, 0.35%, and 70.14%, respectively. At 25 °C, the instantaneous-speed curve showed more continuous fluctuations; at 35 °C, sustained movement periods were less frequent, although brief speed peaks occurred (Figure 1e). Although brief speed peaks remained at 35 °C, the overall activity level was lower.

### 3.2. Agonistic Behavior of E. sinensis Under Thermal Stress

**Figure 2:**
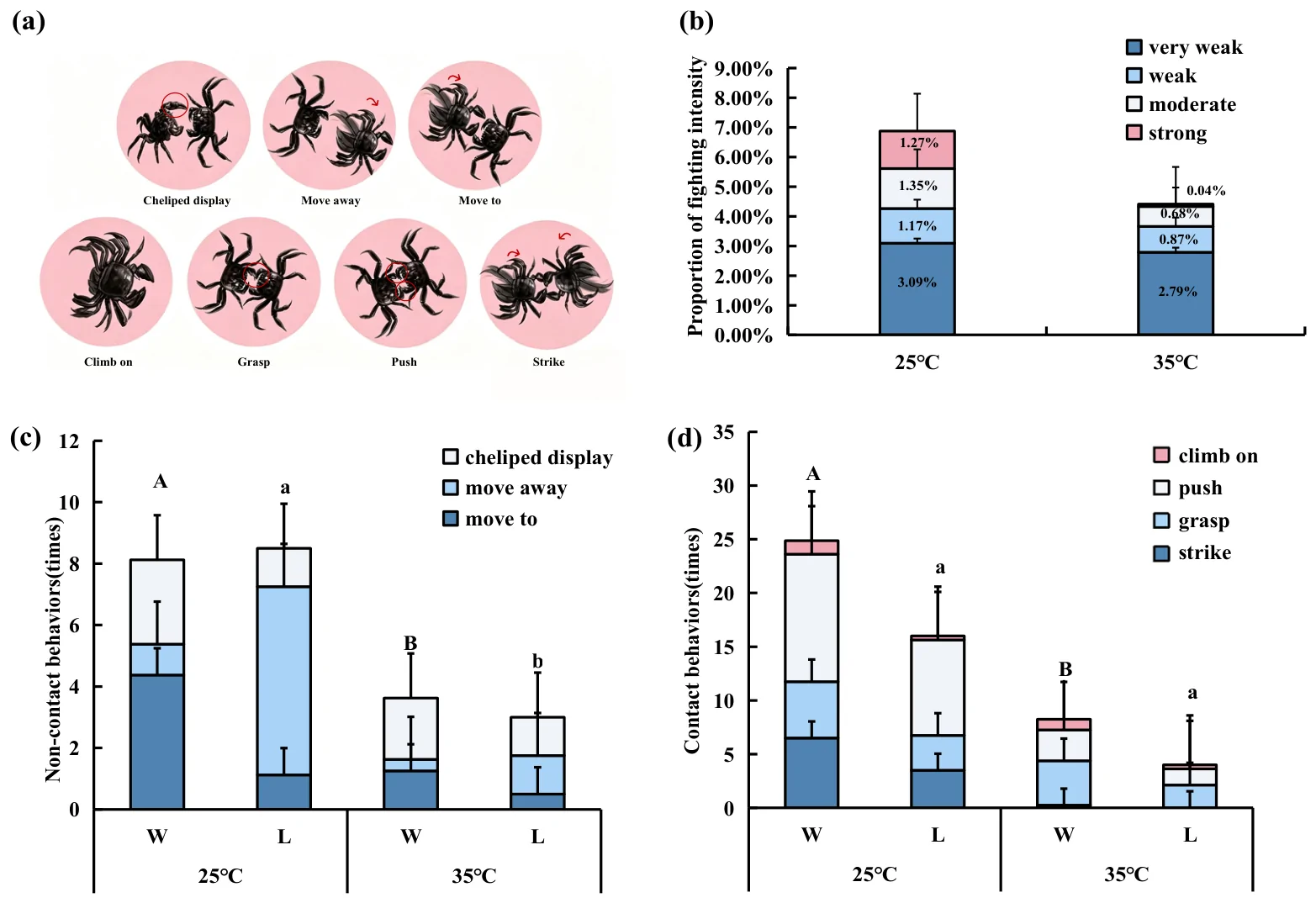
Agonistic behavior of *E. sinensis* under thermal stress. (**a**) Schematic illustration of agonistic behavioral elements in *E. sinensis*; (**b**) proportion of fighting-intensity levels; (**c**) frequency of non-contact behaviors; (**d**) frequency of contact behaviors. In (**a**), red circles highlight the chelipeds or contact regions involved in the illustrated behaviors, and red arrows indicate the direction of movement. W, winner; L, loser. Data are presented as mean ± SD (*n* = 20). Error bars indicate the SD for each fighting-intensity level and behavioral element. Behavioral frequencies were analyzed using generalized linear models. Different uppercase letters indicate significant differences between temperature treatments within the winner group, and different lowercase letters indicate significant differences between temperature treatments within the loser group (*p* < 0.05).

At 25 °C, the four fighting-intensity levels together accounted for 6.88% of the observation period, comprising 3.09% very weak, 1.17% weak, 1.35% moderate, and 1.27% strong interactions (Figure 2b). At 35 °C, the total proportion decreased to 4.38%, comprising 2.79%, 0.87%, 0.68%, and 0.04% very weak, weak, moderate, and strong interactions, respectively. Strong fighting showed the largest reduction. At 25 °C, winners performed more move-to behavior, whereas losers performed more move-away behavior (Figure 2c). Winners and losers both performed significantly fewer non-contact behaviors at 35 °C than at 25 °C (*p* < 0.05). Move-to, move-away, and cheliped-display behaviors all showed lower frequencies at 35 °C. At 25 °C, winners performed more strike, grasp, push, and climb-on behaviors than losers, with strike and push being the most frequent contact behaviors (Figure 2d). The contact-behavior frequency of winners was significantly lower at 35 °C than at 25 °C (*p* < 0.05). Contact behavior also decreased in losers, although the temperature difference was not significant. Winners had a higher contact-behavior frequency than losers at both temperatures.

### 3.3. Energy Metabolism Responses of E. sinensis Under Thermal Stress

**Figure 3:**
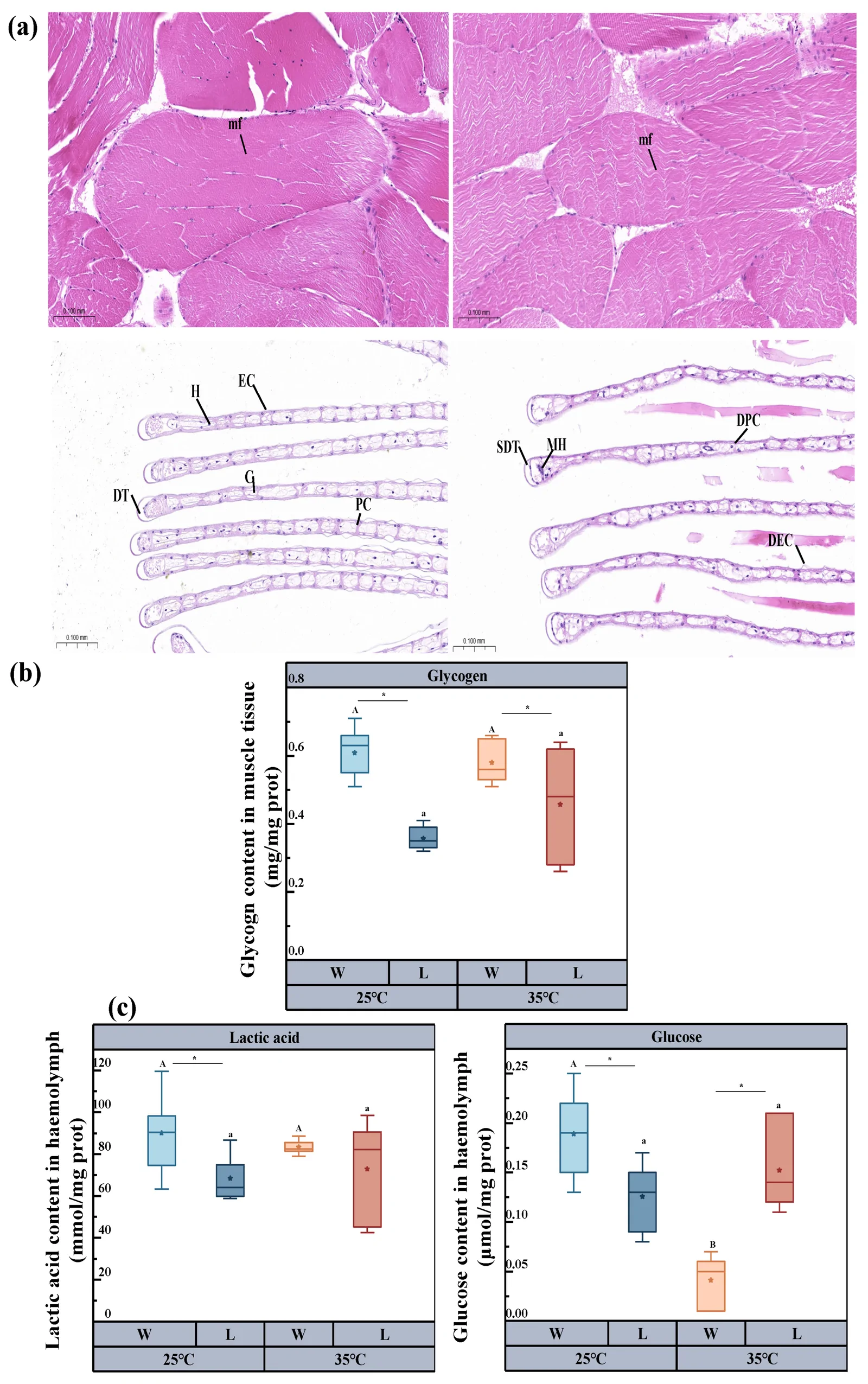
Histological structure and energy substrates of *E. sinensis* under different temperature conditions. (**a**) Representative HE-stained sections of muscle and gill tissues. Muscle tissue shows muscle fibers (mf). Gill tissue shows distal terminus (DT), epithelial cells (EC), hemocytes (H), pillar cells (PC), deformed pillar cells (DPC), swollen distal terminus (SDT), deformed epithelial cells (DEC), and increased hemocytes (MH). (**b**) Glycogen content in muscle tissue; (**c**) lactate and glucose levels in hemolymph. In (**b**,**c**), boxes represent the interquartile range (25th–75th percentiles), horizontal lines within the boxes indicate medians, and star-shaped symbols inside the boxes indicate means. Whiskers indicate the minimum and maximum values. Energy-substrate data are based on four pooled biological samples per group (*n* = 4). W, winner; L, loser. Different uppercase letters indicate significant differences between temperatures within winners, and different lowercase letters indicate significant differences between temperatures within losers; asterisks indicate significant differences between winners and losers at the same temperature (*p* < 0.05).

At 25 °C, the muscle fibers were regularly aligned and closely packed (Figure 3a). At 35 °C, the muscle fibers were disorganized and the interfibrillar spaces were widened. In the gills, the distal termini, epithelial cells, hemocytes, and pillar cells were clearly distinguishable at 25 °C. At 35 °C, the distal termini were swollen, the epithelial and pillar cells were deformed, and greater numbers of hemocytes were observed. Muscle glycogen content was significantly higher in winners than in losers at both 25 °C and 35 °C (*p* < 0.05; Figure 3b). Compared with 25 °C, mean glycogen content changed little in winners and increased in losers at 35 °C; neither temperature-related change was significant. At 25 °C, hemolymph glucose was significantly higher in winners than in losers (*p* < 0.05; Figure 3c). At 35 °C, hemolymph glucose was significantly lower in winners than in losers (*p* < 0.05). Glucose in winners decreased significantly from 25 °C to 35 °C (*p* < 0.05), whereas the numerical increase observed in losers was not significant. Hemolymph lactate was significantly higher in winners than in losers at 25 °C (*p* < 0.05; Figure 3c). At 35 °C, mean lactate was higher in winners than in losers, but the difference was not significant. Lactate did not differ significantly between temperatures within either fighting-outcome group.

**Figure 4:**
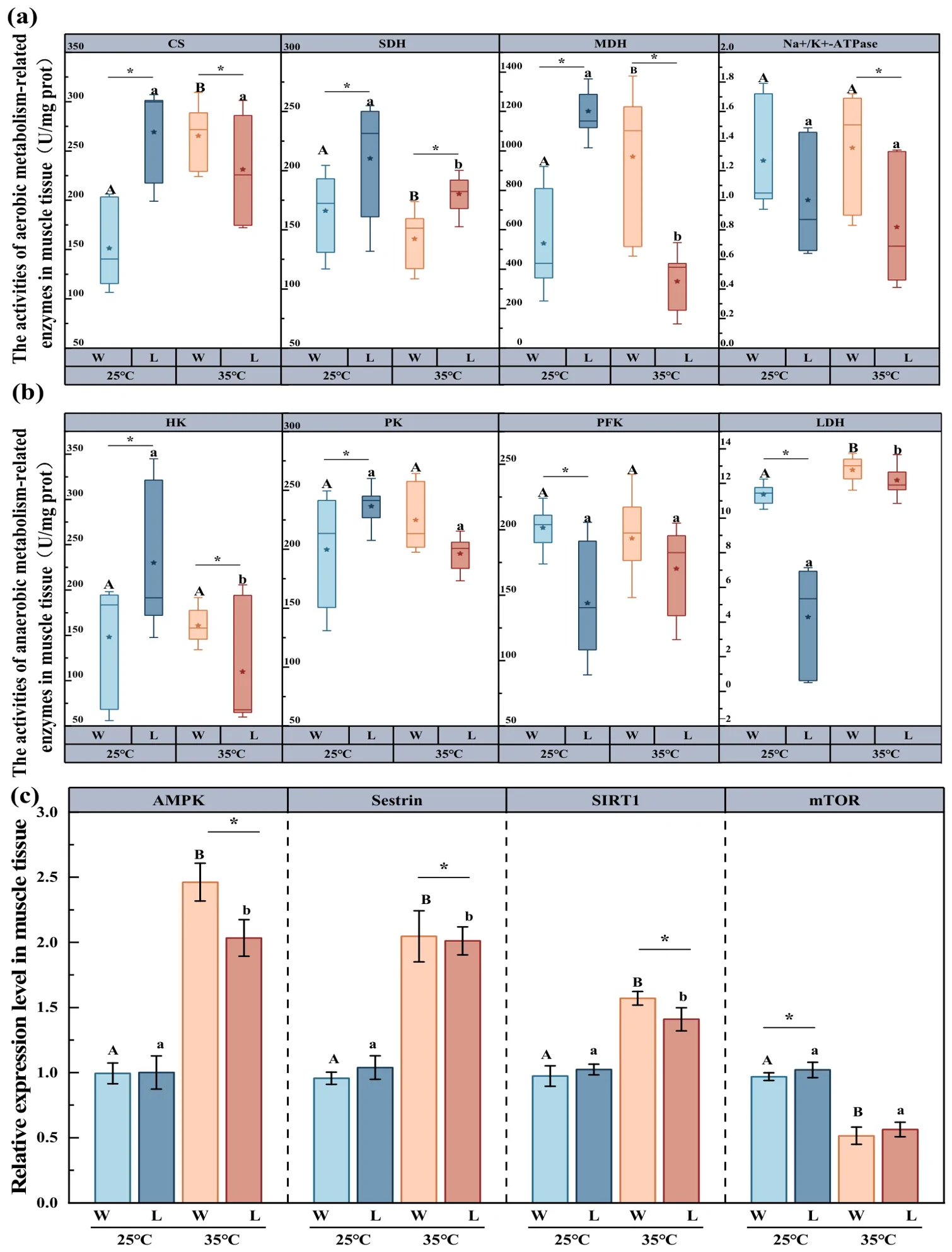
Energy metabolism-related enzyme activities and gene expression in the muscle tissue of *E. sinensis* under different temperature conditions. (**a**) Activities of aerobic metabolism-related enzymes, including citrate synthase (CS), succinate dehydrogenase (SDH), malate dehydrogenase (MDH), and Na^+^/K^+^-ATPase. (**b**) Activities of glycolysis-related enzymes, including hexokinase (HK), pyruvate kinase (PK), phosphofructokinase (PFK), and lactate dehydrogenase (LDH). (**c**) Relative mRNA expression levels of energy-sensing genes *AMPK*, *Sestrin*, *SIRT1*, and *mTOR*. Enzyme activities are expressed as U/mg protein. In (**a**,**b**), boxes represent the interquartile range (25th–75th percentiles), horizontal lines within the boxes indicate medians, and star-shaped symbols inside the boxes indicate means. Whiskers indicate the minimum and maximum values. Data are based on four pooled biological samples per group (*n* = 4). W, winner; L, loser. Effects of temperature, fighting outcome, and their interaction were evaluated by two-way ANOVA followed by Tukey’s multiple-comparison test. Different uppercase letters indicate significant differences between temperatures within winners, and different lowercase letters indicate significant differences between temperatures within losers; asterisks indicate significant differences between winners and losers at the same temperature (*p* < 0.05).

CS activity was lower in winners than in losers at 25 °C (Figure 4a). At 35 °C, CS activity increased in winners but decreased in losers, and the activity in T35W was significantly higher than that in T25W (*p* < 0.05). SDH activity was significantly lower in winners than in losers at both 25 °C and 35 °C (*p* < 0.05). MDH activity was lower in winners than in losers at 25 °C but higher in winners than in losers at 35 °C. Na^+^/K^+^-ATPase activity was higher in winners than in losers at both temperatures, but the difference was significant only at 35 °C (*p* < 0.05). Among the glycolysis-related enzymes, HK activity was significantly lower in winners than in losers at 25 °C (*p* < 0.05) but was higher in winners at 35 °C (Figure 4b). PK activity was lower in winners than in losers at 25 °C but higher in winners than in losers at 35 °C. PFK activity was higher in winners than in losers at both temperatures. LDH activity was significantly higher in winners than in losers at 25 °C (*p* < 0.05) and remained high in both groups at 35 °C. The mRNA levels of *AMPK*, *Sestrin*, and *SIRT1* were significantly higher at 35 °C than at 25 °C in both winners and losers (*p* < 0.05). *mTOR* mRNA levels decreased significantly in winners, whereas the decrease in losers was not significant (Figure 4c). At 35 °C, *AMPK*, *Sestrin*, and *SIRT1* mRNA levels were significantly higher in winners than in losers (*p* < 0.05), whereas *mTOR* mRNA levels did not differ significantly between winners and losers.

**Figure 5:**
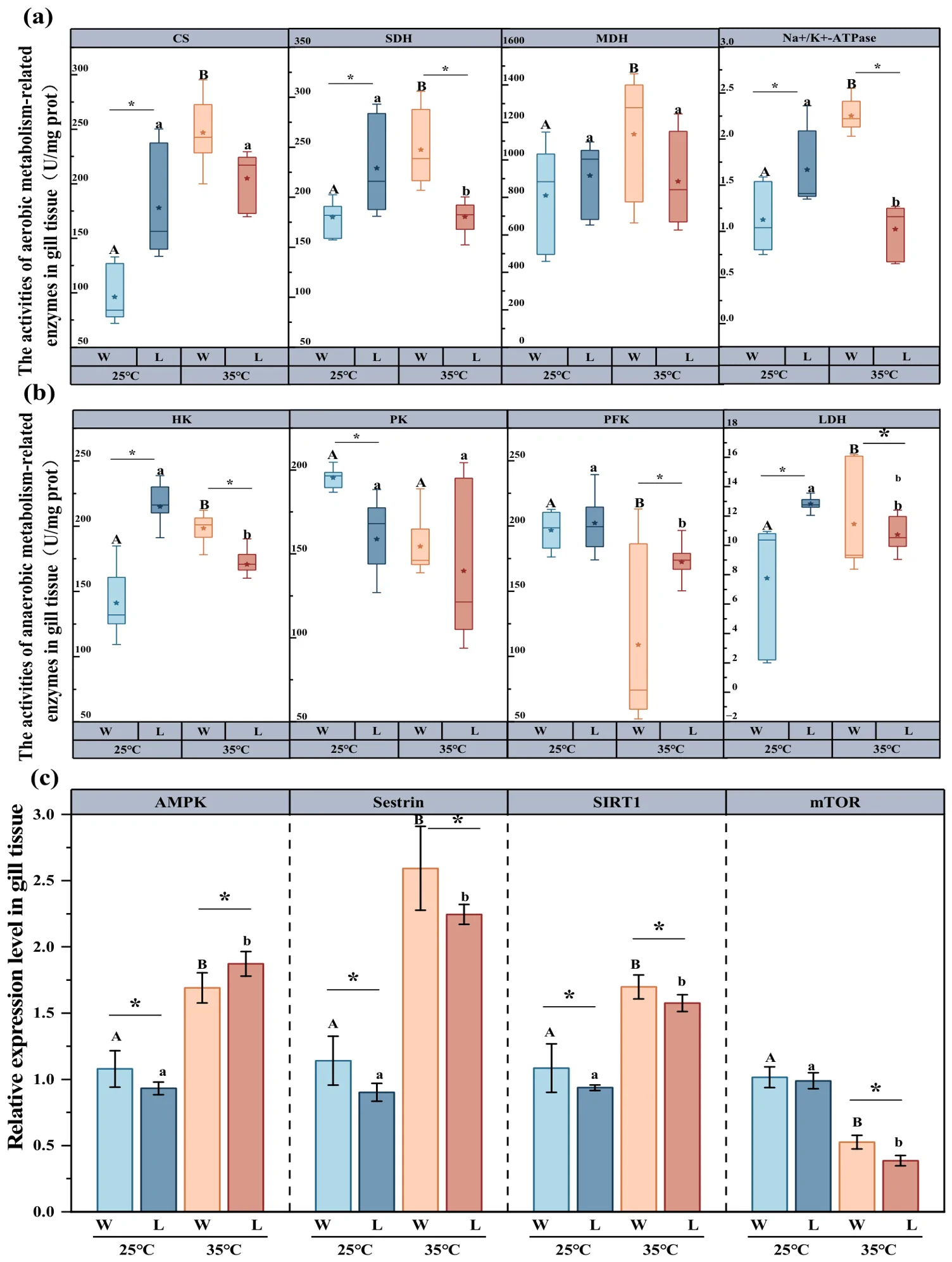
Energy metabolism-related enzyme activities and gene expression in the gill tissue of *E. sinensis* under different temperature conditions. (**a**) Activities of aerobic metabolism-related enzymes, including citrate synthase (CS), succinate dehydrogenase (SDH), malate dehydrogenase (MDH), and Na^+^/K^+^-ATPase. (**b**) Activities of glycolysis-related enzymes, including hexokinase (HK), pyruvate kinase (PK), phosphofructokinase (PFK), and lactate dehydrogenase (LDH). (**c**) Relative mRNA expression levels of energy-sensing genes *AMPK*, *Sestrin*, *SIRT1*, and *mTOR*. Enzyme activities are expressed as U/mg protein. In (**a**,**b**), boxes represent the interquartile range (25th–75th percentiles), horizontal lines within the boxes indicate medians, and star-shaped symbols inside the boxes indicate means. Whiskers indicate the minimum and maximum values. Data are based on four pooled biological samples per group (*n* = 4). W, winner; L, loser. Differences among groups were evaluated by two-way ANOVA followed by Tukey’s multiple-range test. Different uppercase letters indicate significant differences between temperature treatments within winners, and different lowercase letters indicate significant differences between temperature treatments within losers; asterisks indicate significant differences between winners and losers at the same temperature (*p* < 0.05).

CS activity was lower in winners than in losers at 25 °C but higher in winners than in losers at 35 °C (Figure 5a,b). CS activity increased at 35 °C in both groups, with a larger increase in winners. SDH activity was lower in winners than in losers at 25 °C, but this relationship was reversed at 35 °C. At 35 °C, SDH activity increased in winners but decreased in losers. MDH activity was lower in winners than in losers at 25 °C but higher in winners than in losers at 35 °C. Na^+^/K^+^-ATPase activity was lower in winners than in losers at 25 °C, but higher at 35 °C; differences were significant (*p* < 0.05). HK activity was lower in winners than in losers at 25 °C, and this difference was smaller at 35 °C (Figure 5b). PK activity decreased at 35 °C in both groups. PFK activity was lower in winners than in losers at 25 °C and decreased further in winners at 35 °C. LDH activity was lower in winners than in losers at 25 °C but higher in winners than in losers at 35 °C. At 35 °C, *AMPK*, *Sestrin*, and *SIRT1* mRNA levels were higher and *mTOR* mRNA levels were lower than at 25 °C in both winners and losers (Figure 5c). At 35 °C, *AMPK* mRNA levels were significantly higher in losers than in winners, whereas *Sestrin*, *SIRT1*, and *mTOR* mRNA levels were significantly higher in winners than in losers (*p* < 0.05).

### 3.4. Untargeted Metabolomic Responses of E. sinensis Under Thermal Stress and Different Fighting Outcomes

**Figure 6:**
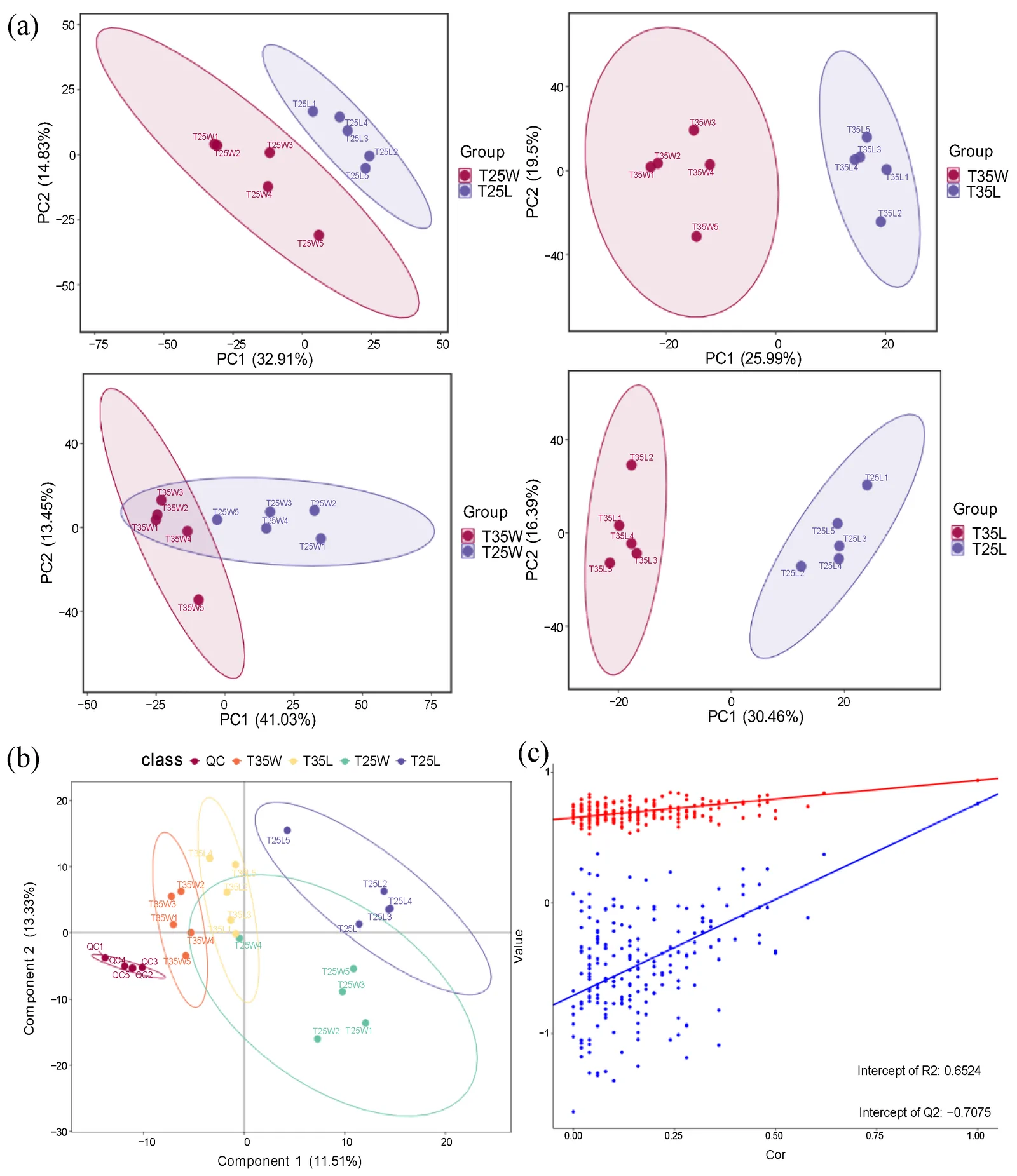
Multivariate statistical analysis of the untargeted metabolomic profiles of *E. sinensis* under different temperatures and fighting outcomes. (**a**) Principal component analysis (PCA) score plots for T25W versus T25L, T35W versus T35L, T35W versus T25W, and T35L versus T25L, from left to right. (**b**) Partial least-squares discriminant analysis (PLS-DA) score plot of all experimental and quality-control (QC) samples. Each experimental point represents one pooled biological sample (*n* = 5 per group); QC points represent quality-control samples. The model yielded R^2^ = 0.9384 and Q^2^ = 0.7602. (**c**) Results of the 200-permutation test for the PLS-DA model. Red and blue points represent R^2^ and Q^2^ values, respectively. The red and blue lines represent the corresponding linear regression fits for R^2^ and Q^2^, respectively. the R^2^ and Q^2^ intercepts were 0.6524 and −0.7075. T25W and T25L represent winners and losers at 25 °C, respectively; T35W and T35L represent winners and losers at 35 °C, respectively.

Multivariate statistical analysis of the muscle metabolomic profiles of *E. sinensis* under different temperatures and fighting outcomes is shown in Figure 6. T25W and T25L were separated mainly along PC1, although their confidence regions partially overlapped, whereas T35W and T35L showed clearer separation. T35W and T25W occupied different regions with partial overlap, whereas T35L and T25L were clearly separated. The PLS-DA score plot also separated the four experimental groups, although some samples overlapped. The QC samples formed a compact cluster. The PLS-DA model yielded R^2^ = 0.9384 and Q^2^ = 0.7602. The 200-permutation test yielded R^2^ and Q^2^ intercepts of 0.6524 and −0.7075, respectively. The first two principal components accounted for 47.74% (T25W versus T25L), 45.44% (T35W versus T35L), 56.36% (T35W versus T25W), and 47.39% (T35L versus T25L) of the total variation, respectively.

**Figure 7:**
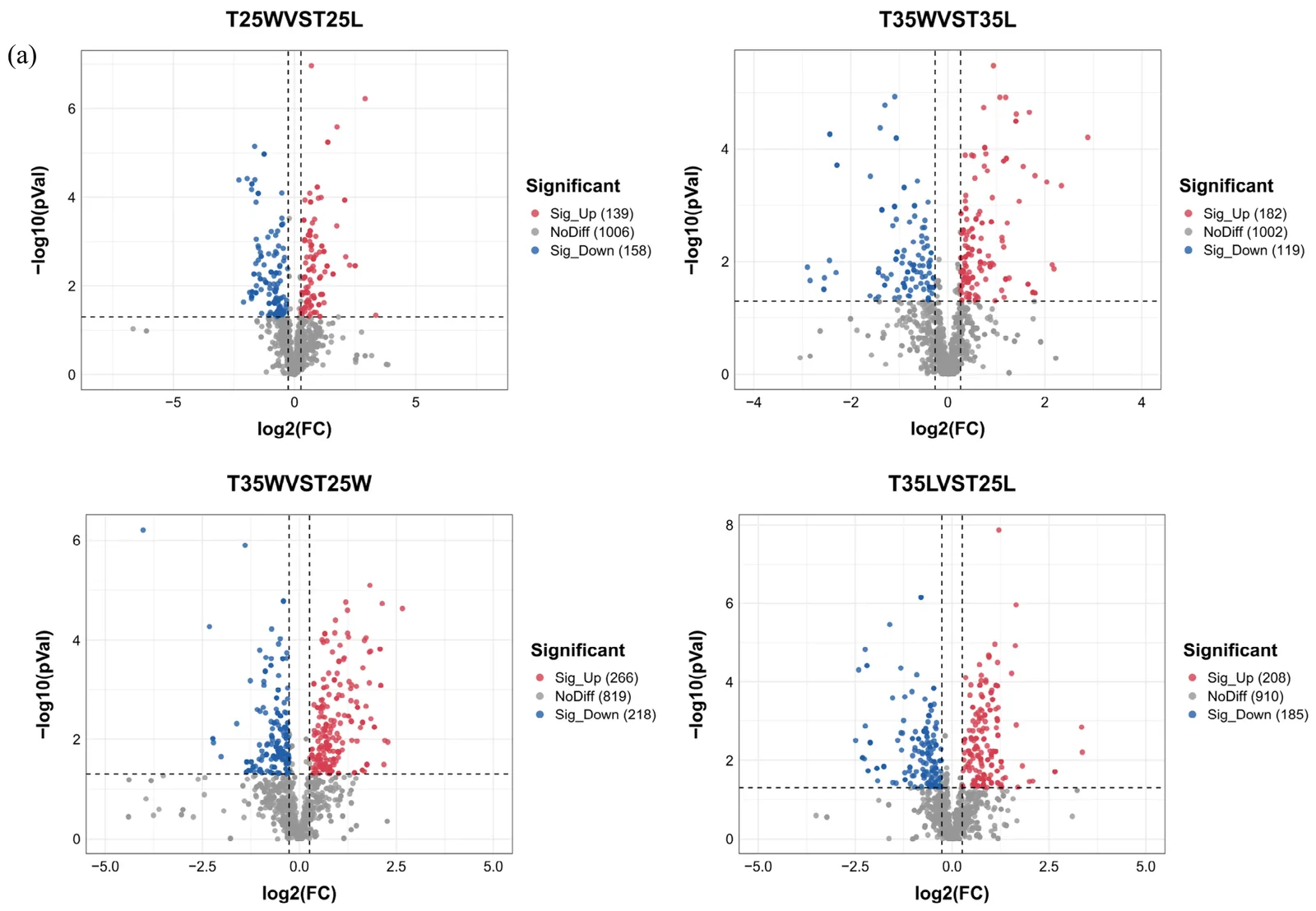

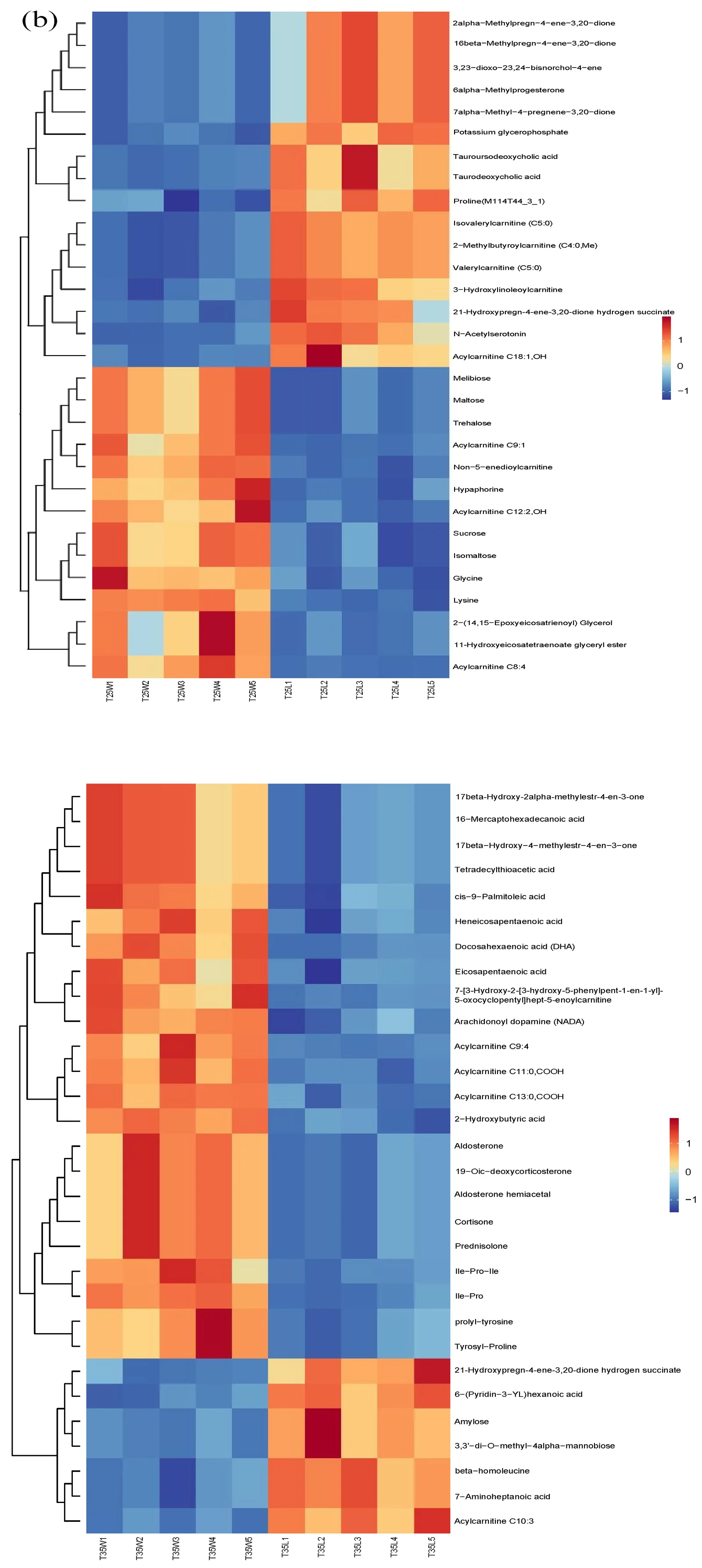

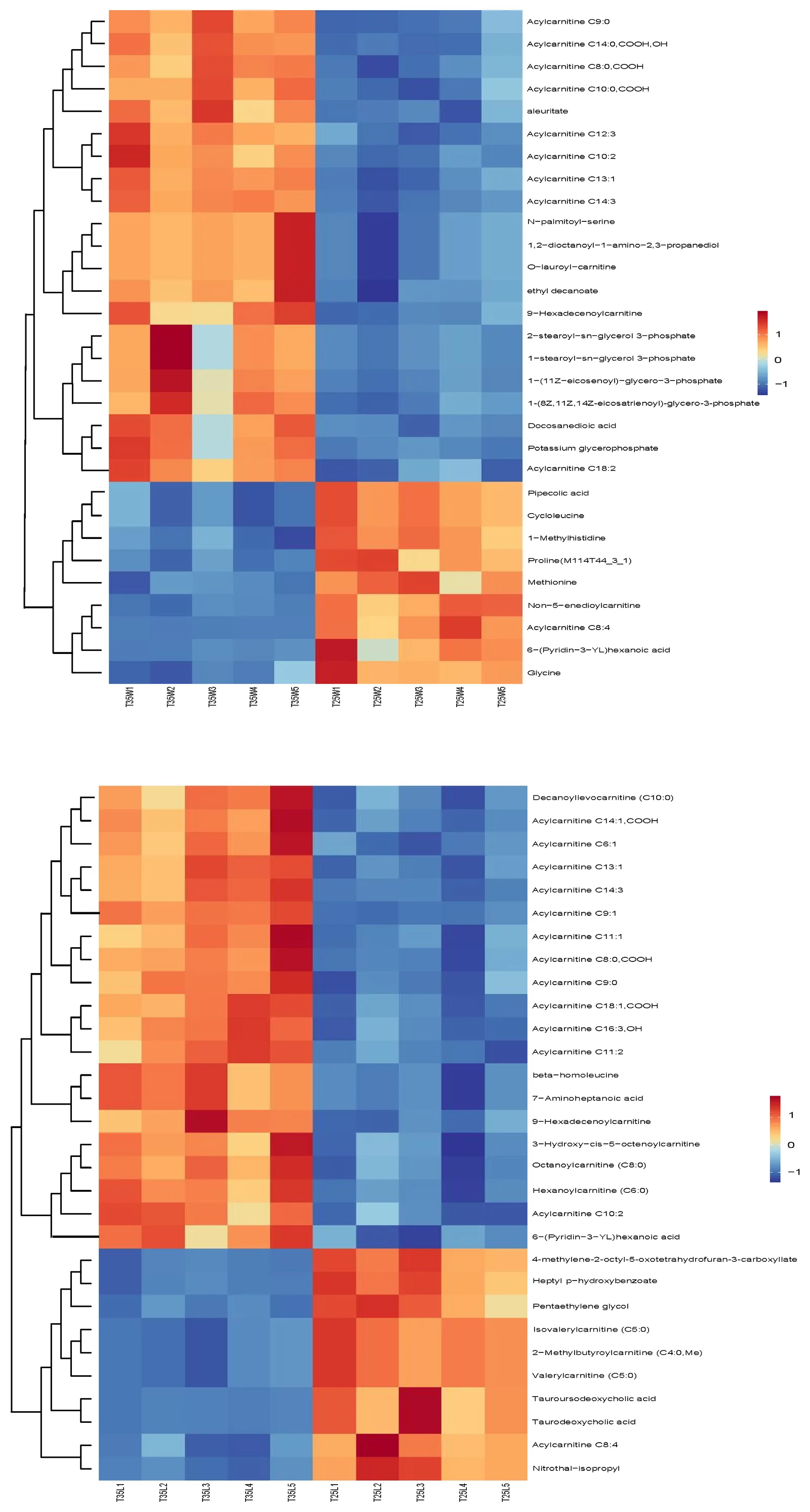
Identification and hierarchical clustering of differential metabolites in *E. sinensis* under different temperatures and fighting outcomes. (**a**) Volcano plots of differential metabolites in T25W versus T25L, T35W versus T35L, T35W versus T25W, and T35L versus T25L. Red, blue, and grey points indicate significantly higher-abundance, significantly lower-abundance, and non-significantly changed metabolites, respectively. The vertical and horizontal dashed lines indicate the fold-change and *p*-value thresholds used for screening differential metabolites. (**b**) Hierarchical clustering heatmaps of differential metabolites in the four corresponding comparisons. Each column represents one pooled biological sample (*n* = 5 per group), and each row represents one metabolite. The black branching lines on the left of each heatmap represent a hierarchical clustering dendrogram, which groups metabolites according to the similarity of their abundance patterns across samples. Red indicates a relatively high metabolite abundance, whereas blue indicates a relatively low abundance. T25W and T25L represent winners and losers at 25 °C, respectively; T35W and T35L represent winners and losers at 35 °C, respectively. Each heatmap displays 30 selected differential metabolites.

T25W versus T25L contained 297 differential metabolites, of which 139 were more abundant and 158 were less abundant in T25W (Figure 7a). T35W versus T35L contained 301 differential metabolites, of which 182 were more abundant and 119 were less abundant in T35W. T35W versus T25W contained 484 differential metabolites, of which 266 were more abundant and 218 were less abundant in T35W. T35L versus T25L contained 393 differential metabolites, of which 208 were more abundant and 185 were less abundant in T35L. Hierarchical clustering revealed distinct abundance patterns in each comparison, and samples from the same temperature and fighting-outcome group generally clustered together (Figure 7b).

The selected differential metabolites displayed in the heatmaps showed comparison-specific abundance patterns. Relative to T25L, T25W showed higher relative abundances of trehalose, maltose, sucrose, glycine, and lysine. Relative to T35L, T35W showed higher relative abundances of docosahexaenoic acid (DHA), eicosapentaenoic acid (EPA), cis-9-palmitoleic acid, and several acylcarnitines. Compared with T25W, T35W showed higher relative abundances of DHA and several long-chain acylcarnitines, but lower relative abundances of glycine, methionine, proline, and pipecolic acid. The T35L versus T25L comparison was likewise characterized by higher relative abundances of multiple acylcarnitines in T35L (Figure 7b).

KEGG annotation assigned the differential metabolites to seven major categories: cellular processes, drug development, environmental information processing, genetic information processing, human diseases, metabolism, and organismal systems (Figure S1). Metabolism contained the largest number of annotated differential metabolites in all four comparisons. Within this category, relatively large numbers of differential metabolites were assigned to metabolic pathways, biosynthesis of secondary metabolites, microbial metabolism in diverse environments, biosynthesis of amino acids, nucleotide metabolism, and biosynthesis of cofactors. Differential metabolites were also assigned to ABC transporters, neuroactive ligand–receptor interaction, the phosphotransferase system, aminoacyl-tRNA biosynthesis, protein digestion and absorption, and lipid-metabolism-related pathways. T35W versus T25W contained the largest number of differential metabolites assigned to general metabolic pathways.

**Figure 8:**
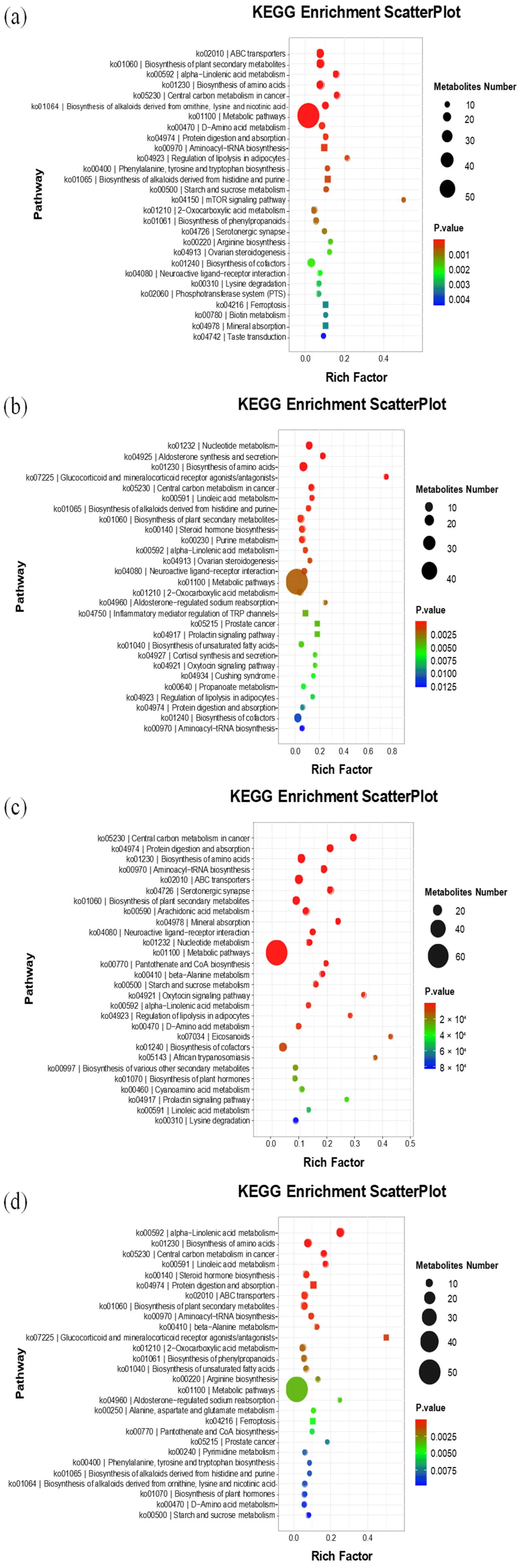
Kyoto Encyclopedia of Genes and Genomes (KEGG) pathway enrichment analysis of differential metabolites in *E. sinensis*. Enrichment bubble plots are shown for (**a**) T25W versus T25L, (**b**) T35W versus T35L, (**c**) T35W versus T25W, and (**d**) T35L versus T25L. The horizontal axis indicates the rich factor, bubble size represents the number of differential metabolites annotated to each pathway, and bubble color represents the enrichment *p* value. Smaller *p* values indicate greater statistical significance of enrichment. T25W and T25L represent winners and losers at 25 °C, respectively; T35W and T35L represent winners and losers at 35 °C, respectively. Metabolomic analyses were based on five pooled biological samples per group (*n* = 5).

The enrichment analysis showed comparison-specific pathway patterns. T25W versus T25L was enriched in ABC transporters, alpha-linolenic acid metabolism, biosynthesis of unsaturated fatty acids, and aminoacyl-tRNA biosynthesis. T35W versus T35L was enriched in nucleotide, histidine, purine, and sphingolipid metabolism, together with neuroactive ligand–receptor interaction. T35W versus T25W showed enrichment of nucleotide metabolism, ABC transporters, starch and sucrose metabolism, fatty acid biosynthesis, and biosynthesis of unsaturated fatty acids, whereas T35L versus T25L showed enrichment of alpha-linolenic acid metabolism, biosynthesis of amino acids, purine metabolism, pantothenate and CoA biosynthesis, and pyruvate metabolism (Figure 8). These enrichment results were evaluated separately from the numbers of metabolites assigned to the broad KEGG categories.

## 4. Discussion

### 4.1. Effects of Thermal Stress on Swimming Behavior and Aggressive Behavior

Swimming behavior reflects the integrated performance of the nervous, muscular, respiratory, and metabolic systems and is highly sensitive to environmental temperature. In juvenile blue swimmer crabs (*Portunus pelagicus*), swimming behavior activity and thermal tolerance varied markedly across temperature treatments, with high temperatures approaching the upper thermal limit of the species [11]. Temperature also altered the daily locomotor rhythm of fiddler crabs (*Uca pugilator*), demonstrating that crustacean activity is closely linked to thermal conditions and seasonal acclimation [26]. In post-larval American lobsters (*Homarus americanus*), warmer water reduced the time spent actively swimming, suggesting that swimming behavior activity may be suppressed when the energetic cost of movement increases [27]. Similarly, elevated temperature reduced active swimming and increased energetic demands in post-larval spiny lobsters [28]. In the present study, exposure to 35 °C reduced the total distance moved, mean swimming speed, and the duration of different active swimming states, while increasing inactive time. These studies indicate that high temperature impaired sustained swimming performance in *E. sinensis*. The reduction in sustained swimming observed in the present study is consistent with a general thermal response pattern in decapod crustaceans.

Agonistic behavior in crustaceans can change in frequency, duration, and intensity in response to changes in environmental conditions [13,29]. Previous studies have shown that increasing environmental temperature from 16 °C to 32 °C increased fighting duration, intensity, and behavioral frequency in the swimming crab *Portunus trituberculatus* [13]. Similarly, agonistic interactions in the shore crab *Carcinus maenas* tended to become more frequent as environmental temperature increased [29]. In the present study, exposure to 35 °C shortened fighting time and reduced the proportion of strong interactions in *E. sinensis*, with non-contact behaviors decreasing in both winners and losers and contact behaviors decreasing significantly in winners. The decrease in non-contact behaviors suggests that high temperature weakened the early phase of agonistic interaction, while the reduction in contact and strong interactions suggests that high temperature also limited the escalation of fights. This pattern suggests that acute thermal stress reduced the overall expression of fighting behavior in *E. sinensis*. Reduced fighting duration and behavioral escalation may serve as behavioral indicators of acute thermal stress in *E. sinensis*, providing a scientific basis for temperature management and welfare assessment in crab aquaculture.

The reduction in both swimming and agonistic behavior shows that thermal stress affected more than one type of behavior in *E. sinensis*. Swimming reflects general locomotor capacity, whereas agonistic behavior reflects competitive performance [11,30]. In the present study, the decline in these two behaviors suggests that 35 °C affected both spontaneous movement and energetically demanding social interaction.

### 4.2. Effects of Thermal Stress on Tissue Structure and Energy Metabolism in E. sinensis

Behavioral changes caused by thermal stress are often associated with structural alterations in muscle tissues involved in locomotion and gill tissues involved in respiration. Pan et al. [20] reported heat-induced structural damage in cardiac tissue of male *E. sinensis* after exposure to 35 °C. Jiang et al. [31] reported distorted and fractured gill filaments, epithelial detachment, cellular swelling, vacuolation, and nuclear abnormalities in heat-sensitive mitten crabs exposed to high temperature. In the present study, at 35 °C, muscle fibers became disorganized and interfibrillar spaces widened, while gill distal regions showed swelling, epithelial deformation, pillar-cell damage, and increased hemocyte accumulation. These structural alterations may be associated with the behavioral and metabolic responses observed under thermal stress.

Stored energy substrates provide the metabolic basis for agonistic behavior because fighting depends on their rapid mobilization and utilization [32]. In *P. trituberculatus*, increasing temperature was accompanied by greater fighting intensity, longer fighting duration, and higher frequencies of agonistic behavior, while fighting was associated with decreased muscle glycogen and increased hemolymph glucose and lactate. During fighting in *P. trituberculatus*, winners showed higher glycolytic and aerobic metabolic activity than losers, indicating that energy-substrate utilization is closely related to contest performance [13]. The expression of agonistic behavior can also be affected by feeding strategy, which changes glycogen and glucose levels and further influences fighting behavior [33]. In the present study, winners contained more muscle glycogen than losers at both temperatures, indicating that individuals that won contests retained higher carbohydrate reserves at the time of sampling. At 35 °C, the relationship between hemolymph glucose and contest outcome changed, whereas lactate showed no consistent temperature effect. These results suggest that thermal stress altered the association between fighting outcome and energy-substrate use in *E. sinensis*, mainly through changes in glycogen reserves and glucose regulation. Therefore, we speculate that the reduction in fighting behavior of *E. sinensis* at 35 °C was closely associated with changes in glycogen storage and glucose regulation.

Changes in the activities of key energy-metabolism enzymes can reflect the patterns of energy utilization in *E. sinensis* under thermal stress. CS, SDH, and MDH participate in the tricarboxylic acid cycle and energy production; HK, PFK, and PK regulate glycolysis; LDH is associated with anaerobic energy supply; and Na^+^/K^+^-ATPase reflects the energy expenditure required for ion transport. Thermal stress can therefore alter the balance between energy production and energy expenditure in crustaceans [13]. Previous studies have shown that in *Litopenaeus vannamei*, HK, PK, and LDH activities increased during the early stage of thermal stress and subsequently decreased, whereas SDH activity initially declined and then recovered toward its initial level [34]. Similarly, exposure to 30 °C inhibited key energy-metabolism processes in adult *E. sinensis*, indicating that severe thermal stress reduced its energy-supply capacity [5]. Consistent with these findings, acute exposure of the Japanese mantis shrimp *Oratosquilla oratoria* to 30 °C reduced MDH-associated energy metabolism during the early stage of stress [35]. In the present study, the activities of key enzymes involved in energy metabolism showed different responses to temperature and fighting outcome. At 35 °C, muscle SDH activity decreased, with a more pronounced reduction in losers, whereas gill CS activity increased. The decrease in muscle SDH activity suggests that energy production for muscle activity was restricted, which is consistent with the observed reductions in swimming and fighting behavior. However, the increase in gill CS activity may reflect enhanced energy production to maintain respiratory and ion-regulatory functions under thermal stress. Collectively, these results suggest that exposure to 35 °C affected multiple energy-metabolic processes in *E. sinensis*, as reflected by the different responses of enzymes involved in aerobic metabolism, glycolysis, anaerobic energy supply, and ion regulation. The greater reduction in SDH activity in losers further suggests that insufficient energy supply may contribute to their poorer competitive performance under thermal stress.

AMPK signaling pathways are central regulators of cellular energy sensing and are closely involved in the adjustment of energy metabolism and behavioral performance under environmental stress [36]. *AMPK* and *SIRT1* jointly participate in the maintenance of cellular energy homeostasis, whereas *Sestrin* can stimulate AMPK-related signaling and suppress *mTOR* under stress [37,38]. In the rock crab *Cancer irroratus*, high temperature increased *AMPK* activity and prolonged exposure also increased *AMPK* mRNA expression, indicating that *AMPK* is involved in the thermal response of crustaceans [39]. Similarly, thermal stress significantly reduced the cardiac expression of several mTOR-pathway genes, including *Rheb*, *S6K*, and *mTOR*, in juvenile Dungeness crabs [40]. This pattern is consistent with the upregulation of *AMPK* and downregulation of *mTOR* observed in the present study. In the present study, *AMPK*, *Sestrin*, and *SIRT1* transcripts were upregulated in muscle and gill tissues at 35 °C, whereas the transcript designated *mTOR* was downregulated. This result suggests that acute high temperature changed the expression of genes related to energy regulation in *E. sinensis*. The higher expression of *AMPK* and *SIRT1* in the muscle of winners suggests that enhanced energy regulation may have facilitated energy allocation to muscle activity, thereby influencing fight outcomes.

### 4.3. Untargeted Metabolomics-Based Analysis of Energy Metabolism and Agonistic Behavioral Responses Under Thermal Stress

Untargeted metabolomics can further reveal how high temperature and fighting outcome are associated with changes in muscle metabolic status. Previous studies have shown that thermal stress affects carbohydrate, amino acid, lipid, and nucleotide metabolism in aquatic invertebrates. In *E. sinensis*, thermal stress altered carbohydrate metabolism, as indicated by increased trehalose content, decreased glycogen content, and downregulated *EsTreh* expression after 24 h of exposure [3]. In *Scapharca subcrenata*, thermal stress affected pathways related to energy, carbohydrate, amino acid, lipid, and nucleotide metabolism; at the metabolite level, aspartic acid and glutamic acid decreased during the early stage of exposure, whereas adenine, cytosine, adenosine, and cytidine monophosphate increased at different exposure times [41]. Similarly, thermal stress altered carbohydrate- and amino-acid-related metabolism in *Chlamys farreri*, with pyruvic acid, glycine, and citric acid identified among the potential metabolic biomarkers [42]. The multivariate analyses revealed differences in muscle metabolic profiles among the four experimental groups. Together with the behavioral, energy-metabolic, and gene-expression results, these patterns support associations between thermal exposure, fighting outcome, and metabolic variation. KEGG enrichment analysis linked the differential metabolites to pathways related to amino acid, nucleotide, and lipid metabolism, as well as aminoacyl-tRNA biosynthesis, ABC transporters, and neuroactive ligand–receptor interaction. These pathways are closely related to energy-substrate use, muscle function, metabolite transport, membrane stability, and behavioral regulation. Therefore, the observed differences in energy metabolism-related pathways suggest that exposure to 35 °C altered the energy metabolic status of *E. sinensis* and may have affected energy allocation to behavior. These metabolite and pathway findings are exploratory, and future studies incorporating FDR correction and independent validation could provide stronger support for their interpretation.

At the metabolite level, the higher relative abundances of trehalose, maltose, sucrose, glycine, and lysine in T25W than in T25L indicate that fighting outcome was accompanied by different carbohydrate- and amino-acid-related metabolic states at 25 °C. Trehalose is a major circulating carbohydrate in crustaceans and is involved in energy metabolism and stress responses [3]; therefore, its higher abundance in T25W may indicate greater availability or retention of carbohydrate reserves in winners after fighting. At 35 °C, the higher relative abundances of DHA, EPA, cis-9-palmitoleic acid, and several acylcarnitines in winners indicate that the metabolic distinction between fighting outcomes extended to unsaturated-fatty-acid- and fatty-acid-transport-related metabolites. Moreover, the increase in DHA and several long-chain acylcarnitines, together with the decrease in glycine, methionine, proline, and pipecolic acid in T35W relative to T25W, was consistent with the simultaneous enrichment of fatty-acid-related, amino-acid-related, and nucleotide-metabolism pathways. Several acylcarnitines were also more abundant in T35L than in T25L, indicating that temperature-associated changes in fatty-acid-transport-related metabolites occurred in both winners and losers. Overall, the winner–loser difference at 25 °C was mainly characterized by carbohydrate- and amino-acid-related metabolites, whereas the corresponding difference at 35 °C involved more unsaturated fatty acids and acylcarnitines. This shift indicates that high temperature changed the types of energy-related metabolites associated with fighting outcome and altered the balance among carbohydrate, amino acid, and lipid metabolism in muscle.

These metabolomic changes further support the relationship between high temperature, reduced agonistic behavior, and altered energy metabolism in *E. sinensis*. At 35 °C, swimming activity, total fighting time, and strong interactions decreased, while the muscle metabolome showed differences in metabolites associated with energy use and behavioral regulation. Therefore, we speculate that the reduction in fighting behavior under high temperature was associated with changes in muscle metabolic state, especially pathways related to energy-substrate use, metabolite transport, and neuroactive regulation.

### 4.4. Integrated Interpretation of Behavioral and Energy-Metabolic Responses in E. sinensis Under Thermal Stress

Overall, the results demonstrate that exposure to 35 °C suppressed swimming and agonistic behavior in *E. sinensis* and that this behavioral inhibition was associated with tissue injury and disturbances in energy metabolism. Previous studies have similarly shown that elevated temperature can affect behavior and energy allocation in aquatic ectotherms, while fighting outcomes are associated with differences in energy status between winners and losers [13,43,44]. In the present study, reduced swimming activity, contact behavior, and fighting intensity at 35 °C were accompanied by gill and muscle injury, altered energy-substrate levels and metabolic enzyme activities, and increased expression of the energy-sensing genes *AMPK*, *Sestrin*, and *SIRT1*. The histological observations provide complementary morphological evidence for the behavioral and energy-metabolic responses to thermal stress. Metabolomic analysis further revealed differences in muscle metabolic profiles associated with temperature and fighting outcome, particularly in pathways related to amino acid, nucleotide, and lipid metabolism. Winners generally exhibited higher muscle glycogen levels, more frequent contact behavior, and distinct metabolic characteristics compared with losers, indicating that energy reserves and metabolic condition were related to contest performance. However, the behavioral decline observed at 35 °C shows that the physiological advantages associated with winning were insufficient to fully counteract the adverse effects of thermal stress. Taken together, these results indicate that high-temperature-induced behavioral inhibition in *E. sinensis* is an integrated response involving tissue damage, altered energy utilization, energy-sensing regulation, and changes in the muscle metabolome, while the differences between winners and losers reflect their distinct capacities to maintain energy supply and contest performance under thermal stress.

## 5. Conclusions

Acute exposure to 35 °C markedly reduced swimming activity as well as the duration and escalation of agonistic behavior in *E. sinensis*. This behavioral suppression coincided with marked alterations in energy metabolism. Untargeted metabolomics further revealed differences in metabolites involved in amino acid, nucleotide, and lipid metabolism, as well as metabolite transport. Taken together, these results indicate that heat-induced behavioral suppression is associated with structural alterations in muscle and gill tissues and changes in energy metabolism. As energy-demanding processes, behavioral changes under thermal stress may reflect the underlying adjustments in energy production and utilization. Consequently, behavior may serve as a potential phenotypic indicator to facilitate the early assessment and management of thermal stress in *E. sinensis*. However, this study examined only male *E. sinensis* and relied on qualitative histological assessment; future research will investigate sex-related differences in thermal-stress responses and include quantitative histological evaluation.

## Figures and Tables

**Table 1:** Definitions of aggressive behavioral elements in *E. sinensis*.

Behavior Type	Behavioral Element	Definition
Non-contact behavior	Move to	One crab approached the other crab during interaction.
Non-contact behavior	Move away	One crab moved away from the other crab during aggressive interaction.
Non-contact behavior	Cheliped display	One crab held its chelipeds forward and displayed a threatening posture.
Contact behavior	Strike	One crab hit the other crab with one or both chelipeds.
Contact behavior	Grasp	One crab used its chelipeds to pinch the carapace, chelipeds, or walking legs of the other crab.
Contact behavior	Push	One crab pushed the opponent with its chelipeds.
Contact behavior	Climb on	One crab attempted to climb onto, or successfully climbed onto, the opponent.

**Table 2:** Criteria used to classify fighting intensity in *Eriocheir sinensis*.

Fighting Intensity	Criteria
Very weak	One crab approached and showed aggressive behavior, while the other crab showed submission. No physical contact occurred.
Weak	Both crabs showed aggressive behavior, and body contact occurred. The fight ended when the winner climbed onto the opponent or when the loser retreated continuously.
Moderate	The fight escalated to grasping and pushing. The loser retreated but still showed threat behavior with chelipeds.
Strong	The loser retreated but repeatedly restarted the fight.

**Table 3:** Primer sequences used for RT-qPCR analysis.

Gene	Primer	GenBank Accession Number
*AMPK*	F: 5′-CAATCGTTGACCTCCCAGAA-3′	MK676045.1
	R: 5′-ACTTCCCTTTCCTTCCCAGAG-3′	
*Sestrin*	F: 5′-TCTTGGATGAGCGGTTTACC-3′	XM_050882146.1
	R: 5′-GGTCTTGGCAGGAGCTTTAT-3′	
*SIRT1*	F: 5′-TGGCGGGAATTAAGAACGTC-3′	XM_050831318.1
	R: 5′-GTCACACTGAGAACACTTGG-3′	
*mTOR*	F: 5′-AGAAGCTGCATGACTGGGAC-3′	XM_050855996.1
	R: 5′-CGGTCACACGACACACTGTA-3′	
*β-actin*	F: 5′-GCATCCACGAGACCACTTACA-3′	KM244725.1
	R: 5′-CTCCTGCTTGCTGATCCACATC-3′	

## Data Availability

The original contributions presented in the study are included in the article material. Further inquiries can be directed to the corresponding author.

## Supplementary Materials

The following supporting information can be downloaded at https://www.mdpi.com/article/10.3390/ani16213440/s1: Table S1: Effects of temperature, fighting outcome and their interaction on agonistic-behavior frequencies; Figure S1: KEGG pathway classification and pathway–metabolite networks of differential metabolites in *E. sinensis*.
