# Supplementary material for: Agonistic Behavior and Energy Metabolic Responses of Eriocheir sinensis Under Thermal Stress with Insights from Untargeted Metabolomics
Source: Animals (Basel). 2026 Oct 31;16(21):3440. doi: 10.3390/ani16213440 (PMC13762283; doi:10.3390/ani16213440)
Supplement: Supplementary file 1 [file animals-16-03440-s001.zip › animals-4622499-supplementary.pdf]

Table S1. Effects of temperature, fighting outcome and their interaction on agonistic-behavior frequencies.

Behavioral endpoint	Temperature *P*	Fighting outcome *P*	Temperature × outcome *P*
Non-contact frequency	0.0014	0.6736	0.4843
Contact frequency	2.101×10^{-7}	0.0044	0.4659

P values were obtained from likelihood-ratio tests of a negative-binomial generalized linear mixed model with temperature, fighting outcome and their interaction as fixed effects and contest identity as a random intercept. Centered coding was used for both factors. Non-contact frequency is the sum of move to, move away and cheliped display. Contact frequency is the sum of strike, grasp, push and climb on.

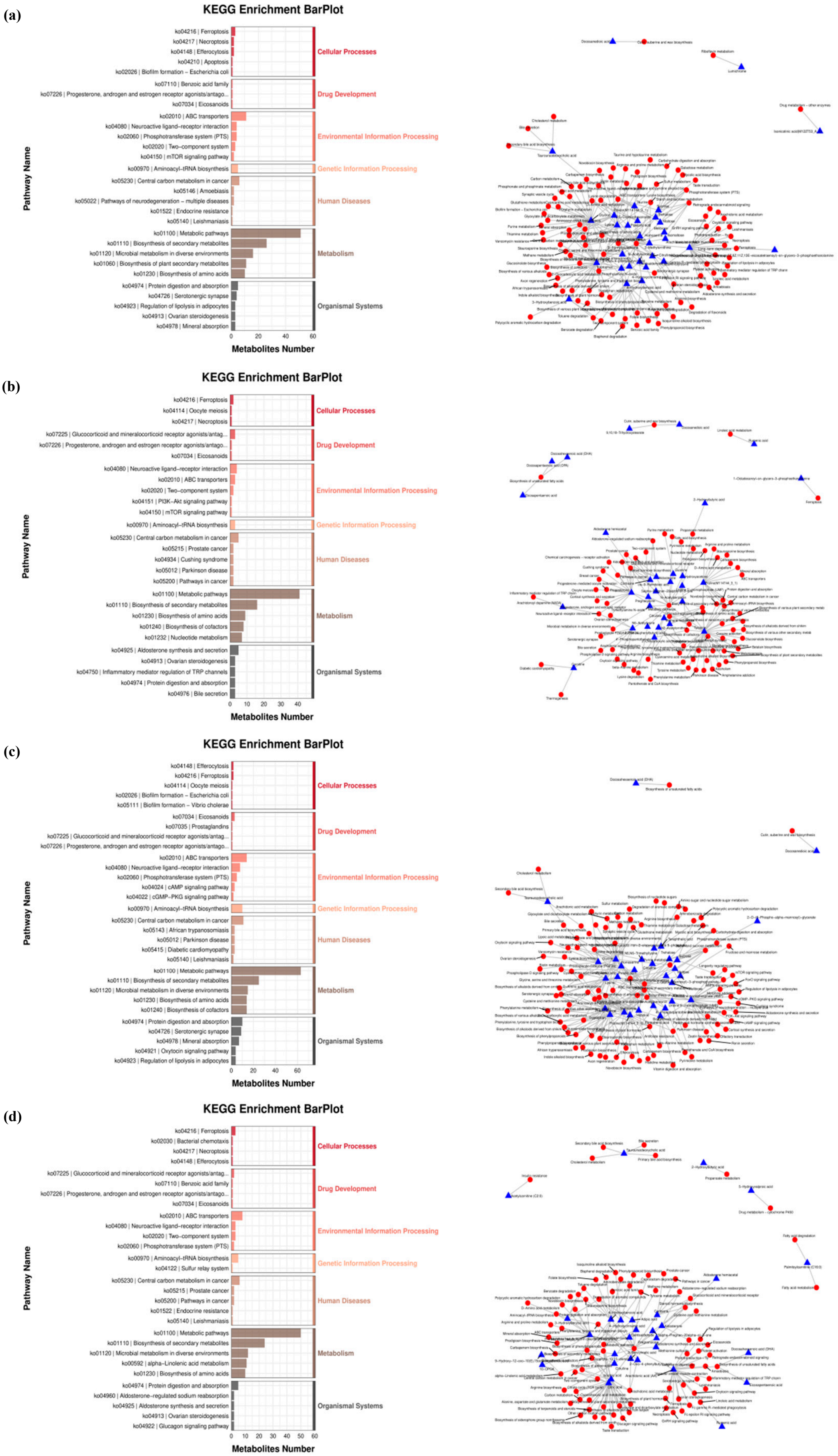
(a)
KEGG Enrichment BarPlot
ko04216 | Ferroptosis
ko04217 | Necroptosis
ko04148 | Efferocytosis
ko04210 | Apoptosis
ko02026 | Biofilm formation – Escherichia coli
ko07110 | Benzoic acid family
ko07226 | Progesterone, androgen and estrogen receptor agonists/antag...
ko07034 | Eicosanoids
ko02010 | ABC transporters
ko04080 | Neuroactive ligand–receptor interaction
ko02060 | Phosphotransferase system (PTS)
ko02020 | Two–component system
ko04150 | mTOR signaling pathway
ko00970 | Aminoacyl–tRNA biosynthesis
ko05230 | Central carbon metabolism in cancer
ko05146 | Amoebiasis
ko05022 | Pathways of neurodegeneration – multiple diseases
ko01522 | Endocrine resistance
ko05140 | Leishmaniasis
ko01100 | Metabolic pathways
ko01110 | Biosynthesis of secondary metabolites
ko01120 | Microbial metabolism in diverse environments
ko01060 | Biosynthesis of plant secondary metabolites
ko01230 | Biosynthesis of amino acids
ko04974 | Protein digestion and absorption
ko04726 | Serotonergic synapse
ko04923 | Regulation of lipolysis in adipocytes
ko04913 | Ovarian steroidogenesis
ko04978 | Mineral absorption
Cellular Processes
Drug Development
Environmental Information Processing
Genetic Information Processing
Human Diseases
Metabolism
Organismal Systems
Pathway Name
0
20
40
60
Metabolites Number
(b)
KEGG Enrichment BarPlot
ko04216 | Ferroptosis
ko04114 | Oocyte meiosis
ko04217 | Necroptosis
ko07225 | Glucocorticoid and mineralocorticoid receptor agonists/antag...
ko07226 | Progesterone, androgen and estrogen receptor agonists/antago...
ko07034 | Eicosanoids
ko04080 | Neuroactive ligand–receptor interaction
ko02010 | ABC transporters
ko02020 | Two–component system
ko04151 | PI3K–Akt signaling pathway
ko04150 | mTOR signaling pathway
ko00970 | Aminoacyl–tRNA biosynthesis
ko05230 | Central carbon metabolism in cancer
ko05215 | Prostate cancer
ko04934 | Cushing syndrome
ko05012 | Parkinson disease
ko05200 | Pathways in cancer
ko01100 | Metabolic pathways
ko01110 | Biosynthesis of secondary metabolites
ko01230 | Biosynthesis of amino acids
ko01240 | Biosynthesis of cofactors
ko01232 | Nucleotide metabolism
ko04925 | Aldosterone synthesis and secretion
ko04913 | Ovarian steroidogenesis
ko04750 | Inflammatory mediator regulation of TRP channels
ko04974 | Protein digestion and absorption
ko04976 | Bile secretion
Cellular Processes
Drug Development
Environmental Information Processing
Genetic Information Processing
Human Diseases
Metabolism
Organismal Systems
Pathway Name
0
10
20
30
40
Metabolites Number
(c)
KEGG Enrichment BarPlot
ko04148 | Efferocytosis
ko04216 | Ferroptosis
ko04114 | Oocyte meiosis
ko02026 | Biofilm formation – Escherichia coli
ko05111 | Biofilm formation – Vibrio cholerae
ko07034 | Eicosanoids
ko07035 | Prostaglandins
ko07225 | Glucocorticoid and mineralocorticoid receptor agonists/antag...
ko07226 | Progesterone, androgen and estrogen receptor agonists/antago...
ko02010 | ABC transporters
ko04080 | Neuroactive ligand–receptor interaction
ko02060 | Phosphotransferase system (PTS)
ko04024 | cAMP signaling pathway
ko04022 | cGMP–PKG signaling pathway
ko00970 | Aminoacyl–tRNA biosynthesis
ko05230 | Central carbon metabolism in cancer
ko05143 | African trypanosomiasis
ko05012 | Parkinson disease
ko05415 | Diabetic cardiomyopathy
ko05140 | Leishmaniasis
ko01100 | Metabolic pathways
ko01110 | Biosynthesis of secondary metabolites
ko01120 | Microbial metabolism in diverse environments
ko01230 | Biosynthesis of amino acids
ko01240 | Biosynthesis of cofactors
ko04974 | Protein digestion and absorption
ko04726 | Serotonergic synapse
ko04978 | Mineral absorption
ko04921 | Oxytocin signaling pathway
ko04923 | Regulation of lipolysis in adipocytes
Cellular Processes
Drug Development
Environmental Information Processing
Genetic Information Processing
Human Diseases
Metabolism
Organismal Systems
Pathway Name
0
20
40
60
Metabolites Number
(d)
KEGG Enrichment BarPlot
ko04216 | Ferroptosis
ko02030 | Bacterial chemotaxis
ko04217 | Necroptosis
ko04148 | Efferocytosis
ko07225 | Glucocorticoid and mineralocorticoid receptor agonists/antag...
ko07110 | Benzoic acid family
ko07226 | Progesterone, androgen and estrogen receptor agonists/antago...
ko07034 | Eicosanoids
ko02010 | ABC transporters
ko04080 | Neuroactive ligand–receptor interaction
ko02020 | Two–component system
ko02060 | Phosphotransferase system (PTS)
ko00970 | Aminoacyl–tRNA biosynthesis
ko04122 | Sulfur relay system
ko05230 | Central carbon metabolism in cancer
ko05215 | Prostate cancer
ko05200 | Pathways in cancer
ko01522 | Endocrine resistance
ko05140 | Leishmaniasis
ko01100 | Metabolic pathways
ko01110 | Biosynthesis of secondary metabolites
ko01120 | Microbial metabolism in diverse environments
ko00592 | alpha–Linolenic acid metabolism
ko01230 | Biosynthesis of amino acids
ko04974 | Protein digestion and absorption
ko04960 | Aldosterone–regulated sodium reabsorption
ko04925 | Aldosterone synthesis and secretion
ko04913 | Ovarian steroidogenesis
ko04922 | Glucagon signaling pathway
Cellular Processes
Drug Development
Environmental Information Processing
Genetic Information Processing
Human Diseases
Metabolism
Organismal Systems
Pathway Name
0
10
20
30
40
50
60
Metabolites Number

Figure S1. KEGG pathway classification and pathway–metabolite networks of differential metabolites in *E. sinensis*.

Comparisons are shown for (a) T25W versus T25L, (b) T35W versus T35L, (c) T35W versus T25W, and (d) T35L versus T25L. Left panels show KEGG pathway classifications; the horizontal axis indicates the number of differential metabolites annotated to each pathway, and colors distinguish major KEGG functional categories. Right panels show pathway–metabolite networks, with red circles representing pathways, blue triangles representing differential metabolites, and connecting lines indicating annotation relationships between metabolites and pathways. T25W and T25L represent winners and losers at 25 °C, respectively; T35W and T35L represent winners and losers at 35 °C, respectively. Analyses were based on five pooled biological samples per group (n = 5).
